# Cyclotriphosphate: A Brief History, Recent Developments, and Perspectives in Synthesis

**DOI:** 10.1002/chem.201904433

**Published:** 2019-12-11

**Authors:** Dominik Bezold, Tobias Dürr, Jyoti Singh, Henning J. Jessen

**Affiliations:** ^1^ Institute of Organic Chemistry University of Freiburg 79104 Freiburg Germany; ^2^ Freiburg Research Institute for Advanced Studies (FRIAS) University of Freiburg 79104 Freiburg Germany; ^3^ Cluster of Excellence livMatS @ FIT—Freiburg Center for, Interactive Materials and Bioinspired Technologies University of Freiburg Georges-Köhler-Allee 105 79110 Freiburg Germany

**Keywords:** labelling, phosphates, phosphorylation, phosphorous, synthetic methods

## Abstract

There has been a recent upsurge in the study and application of approaches utilizing cyclotriphosphate **1** (cyclo‐TP, also known as trimetaphosphate, TMP) and/or proceeding through its analogues in synthetic chemistry to access modified oligo‐ and polyphosphates. This is especially useful in the area of chemical nucleotide synthesis, but by no means restricted to it. Enabled by new high yielding and easy‐to‐implement methodologies, these approaches promise to open up an area of research that has previously been underappreciated. Additionally, refinements of concepts of prebiotic phosphorylation chemistry have been disclosed that ultimately rely on cyclo‐TP **1** as a precursor, placing it as a potentially central compound in the emergence of life. Given the importance of such concepts for our understanding of prebiotic chemistry in combination with the need to readily access modified polyphosphates for structural and biological studies, this paper will discuss selected recent developments in the field of cyclo‐TP chemistry, briefly touch on ultraphosphate chemistry, and highlight areas in which further developments can be expected.

## Graphical abstract

**Cyclotriphosphate (cyclo‐TP) and its analogues**: Selected recent developments in the field of cyclo‐TP chemistry as well as ultraphosphate chemistry are discussed in this Review‐type article.

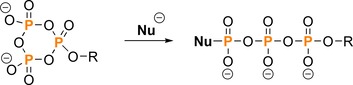

## Introduction

**Figure 1:**
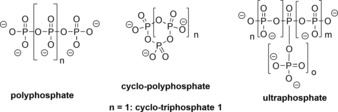
The condensed phosphates.

Condensed phosphates—molecules containing phosphoric anhydride bonds—can occur in linear, cyclic, and branched forms,^1^ giving rise to polyphosphates,^2^ cyclo‐polyphosphates, and ultrapolyphosphates, respectively (Figure 1).^3^ Also, combinations of such substructures have been described or proposed.3b The potential diversity of the condensed phosphates has yet to be explored and arguably the synthetic challenges associated with such an endeavor remain profound given the difficulties in handling such compounds.3c Recent results, however, indicate that many defined condensed phosphates could be readily available following optimized protocols and thus, new types of compounds are becoming available. This will facilitate the design of new reagents^4^ and polyelectrolytes^5^, but also probes and tools to answer fundamental questions of chemistry and biology.^6^ It is, therefore, an exciting time to work in the interdisciplinary field of the condensed phosphates.

**Figure 2:**
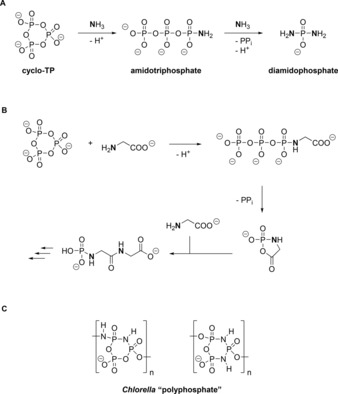
A) Generation of potential prebiotic phosphorylation agents from cyclo‐TP **1**. B) Condensation of amino acids mediated by cyclo‐TP **1**. C) Some proposed Chlorella “polyphosphates” with imido nitrogen atoms.

Water‐soluble polyphosphates (linear and cyclic) are formed under prebiotic conditions as a result of volcanic activity.^7^ Consequently, these compounds could have been available as soluble phosphate sources in prebiotic phosphorylation chemistry and much research has been dedicated to understand phosphorylation reactions based on polyphosphate and cyclo‐TP **1**.^8^ As the direct transformation of nucleosides and sugars with **1** is sluggish under several conditions, it may simply serve as the precursor for other more potent alcohol‐phosphorylating agents obtained by sequential aminolysis, such as amidotriphosphate,^9^ or diamidophosphate^10^ (Figure 2 A).

It is noteworthy that the presence of an amine nucleophile and another nucleophile in one molecule can trigger phosphorylation cascades, such as found in the condensation of amino acids using cyclo‐TP **1** (Figure 2 B). This supports the argument that in prebiotic phosphorylation reactions relying on cyclo‐TP **1**, amine nucleophiles potentially played an important role.^11^ These examples generally assume phosphorous derivatives in the oxidation state P^V^, yet other possibilities include P^III^ derivatives as hydrolysis products of schreibersite, which is delivered by meteorites.^12^ Apart from the potential role of cyclo‐TP **1** in prebiotic phosphorylation chemistry, not much is known about its metabolism in living organisms. Clearly, it has been identified in yeast extracts in the 1950s^13^ but follow‐up studies, mainly dealing with algae, have been scarce.^14^ Also the exciting finding that the single celled green algae *Chlorella* contains cyclic polyphosphates including imido nitrogen atoms^15^ has received only very limited attention (Figure 2 C shows some proposed structures still awaiting validation). Thus, much research remains to be done—not only regarding prebiotic chemistry of cyclo‐TP **1** and related analogues, such as its adenosine ester,^16^ but also about its potential biological functions.

**Scheme 1:**
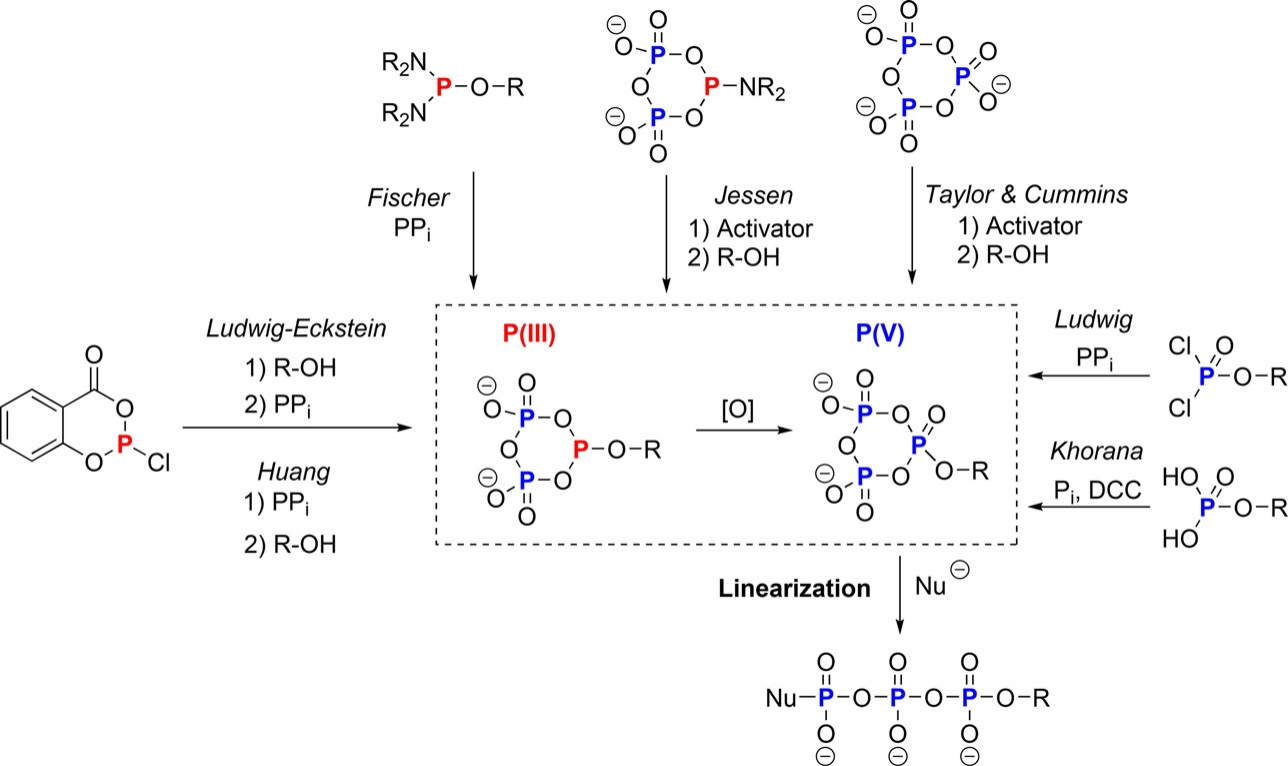
Overview of different strategies to generate modified (deoxy)‐cyclotriphosphates. P_i_=orthophosphate; PP_i_=pyrophosphate; DCC=dicyclohexylcarbodiimide.

Modified cyclo‐TP **1** has inspired the minds of synthetic and structural chemists for a long time. The potential of **1** to directly generate modified triphosphate esters using alcohols is a highly appealing strategy.^17^ The reactions should proceed through a modified cyclo‐TP monoester that is then linearized with water (or other nucleophiles). Despite initial efforts in the 1960s^18^ and 70s,^19^ this reaction has only been transformed into a useful application several decades later. The activation of cyclo‐TP **1** with mesitylene chloride and DABCO^20^ is the first example of a synthetically broadly useful application. Recently, Cummins et al. conducted a landmark study using the peptide‐coupling reagent PyAOP giving stable and isolatable salts of cyclo‐TP esters. Moreover, X‐ray crystal structures of the modified cyclo‐TPs were obtained, and intriguingly, also of the activated species.^21^ These approaches now enable the direct customization of cyclo‐TP **1** with different nucleophiles followed by hydrolysis to give linear modified triphosphates. Of note, modified cyclo‐TPs have been generated synthetically by other successful approaches previously, but not by direct modification of intact cyclo‐TP **1**. An overview of the strategies can be found in Scheme 1, which have also been the subject of comprehensive reviews.^22^

This Concept paper aims to provide an update of how cyclo‐TP **1** can be chemically generated or directly activated focusing on recent approaches. We have arbitrarily grouped the approaches into three categories: 1) generation of cyclo‐TP esters, in which the P‐anhydrides are constructed subsequent to R‐OH modification; 2) generation of cyclo‐TP esters, which rely on direct functionalization of cyclo‐TP **1**; 3) generation of activated cyclo‐TP esters, in which the P‐anhydrides are constructed prior to R‐OH modification. Especially the second and third strategy will be discussed in more detail, because strategy 1 has been known and applied for a long time. We will then discuss under 4) how cyclo‐TP analogues can be used to construct P‐anhydrides as well.

These approaches have been used in their majority to generate modified nucleoside oligophosphates that serve as versatile tools to monitor, perturb, and understand biological processes. Especially, non‐hydrolyzable analogues of nucleoside triphosphates belong to the standard repertoire in structural biology.^23^ This focus is not surprising, given the central roles of nucleoside triphosphates in life.^24^ Yet, the approaches discussed herein are highly useful to access other structures as well, which will be discussed later. Several applications of NTP analogues, such as monitoring enzyme activity using spectroscopic/fluorescent probes or analyzing the (m)RNA cap structure, just to provide two examples of how useful such analogues are, have recently been the subject of two in‐depth Reviews, which are highly recommended to the interested reader.^25^

## 1 Synthesis of Cyclo‐TP Esters Using Phosphoric Anhydride Forming Reactions

**Scheme 2:**
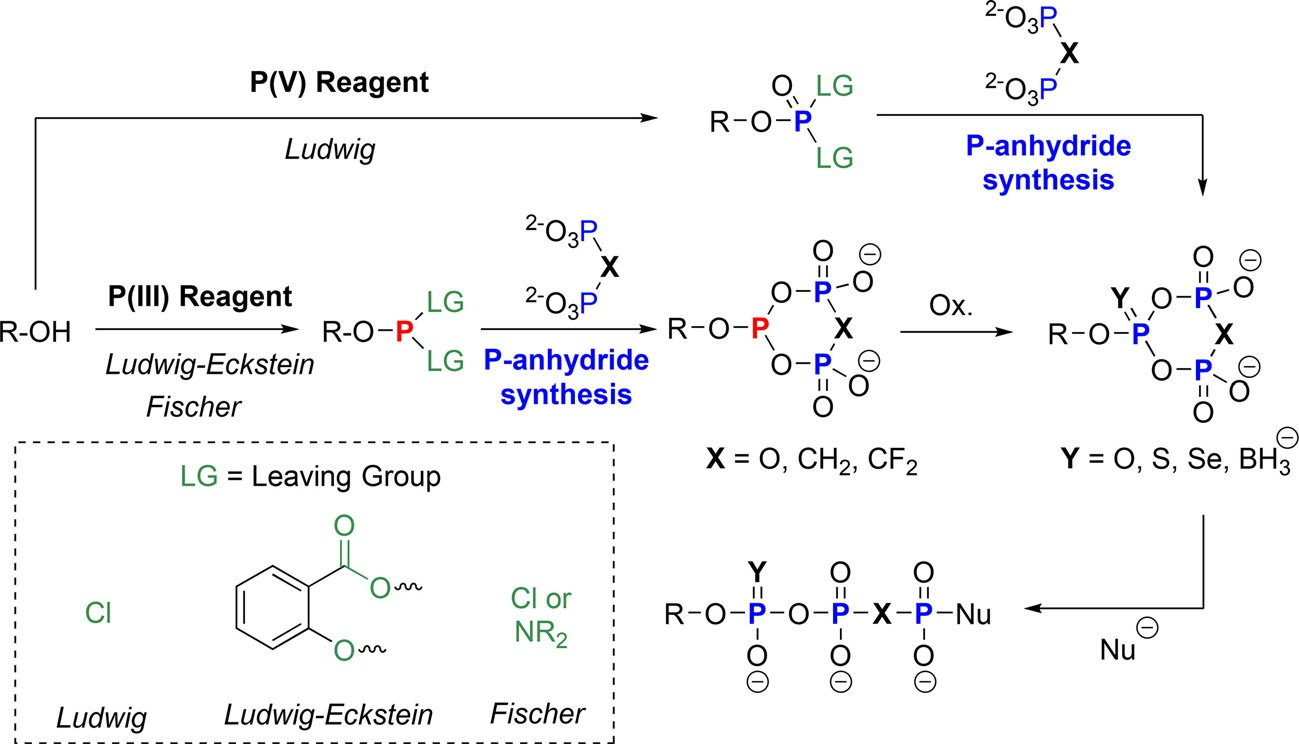
Synthesis of (deoxy)‐cyclo‐TP by formation of phosphoric anhydrides.

Especially the Ludwig–Eckstein protocol has found widespread application.^26^ It proceeds by using the commercial P^III^ reagent 2‐chloro‐1,3,2‐benzodioxaphosphorin‐4‐one, which is initially added to the nucleoside followed by pyrophosphate to generate a deoxy‐cyclo‐TP ester. This intermediate is then oxidized to the cyclo‐TP ester and linearized (Scheme 2).22b, 22e Another approach generating deoxy‐cyclo‐TPs as intermediates was published by Fischer.^27^ In this case, a phosphordiamidite or dichloridite is used as precursor (Scheme 2). The widely‐applied Ludwig–Eckstein approach was preceded by P^V^ chemistry following the Yoshikawa and Ludwig strategies, and earlier protocols by Khorana using dehydrating agents, such as DCC.^28^ The Khorana method likely leads to accumulation of nucleoside triphosphates by a cyclo‐TP ester, which was later demonstrated by ^18^O isotope exchange experiments.^29^ When using P^V^ chemistry, the oxidation step can be omitted, which can be beneficial in cases were labile nucleosides are used. However, the oxidation step can also be highly valuable to introduce additional modifications in the α‐position. Moreover, coordination of BH_3_ enables the generation of boranophosphates, which are useful nucleotide analogues and antivirals.^30^ Also, non‐hydrolyzable analogues can be generated using this approach, for example, bearing CF_2_ replacements of the β–γ oxygen atom (Scheme 2).^31^

## 2 Direct Modification of Cyclo‐TP

**Scheme 3:**
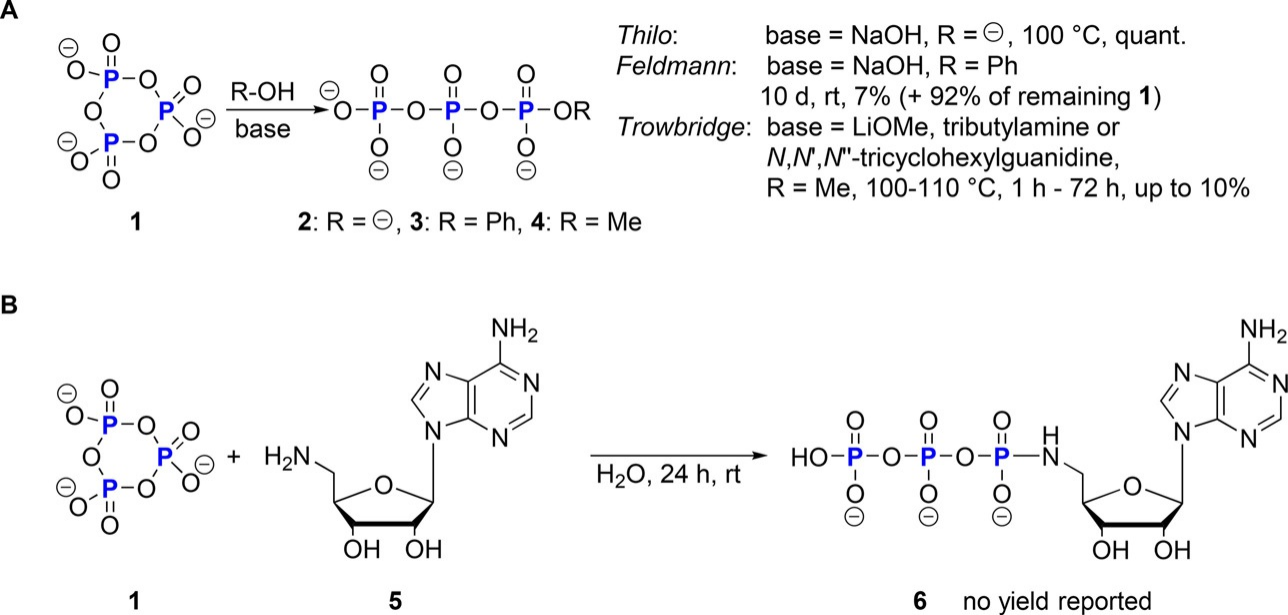
A) First reported linearizations of cyclo‐TP **1**. B) First synthesis of an ATP analogue using cyclo‐TP **1**.

In 1949, Thilo reported that after hydrolysis of cyclo‐TP **1** with sodium hydroxide only linear triphosphate **2** was obtained.^32^ In the following years, various new strategies were published using different amine nucleophiles for the linearization. The first phenolysis of **1** in basic solution (pH 9) was reported by Feldmann in 1966.^18^ The reaction was very slow and after 10 days only 7 % of the linear product **3** was obtained. In 1971, Trowbridge reported the pH dependency of the ring‐opening of **1** using methanol as nucleophile.^19^ If no base is added to the solution of cyclo‐TP **1** in anhydrous methanol, only methylated monophosphate was isolated as product. When adding a base, such as lithium methoxide, up to 10 % of methyl triphosphate **4** was obtained (Scheme 3 A). Additionally, it was possible to synthesize an ATP analogue **6** containing a phosphoramidate at the 5′‐carbon of the ribose by treating the 5′‐amino nucleoside **5** with **1**. This was the first example of applying cyclo‐TP **1** as a reagent in nucleotide chemistry (Scheme 3 B).

**Scheme 4:**
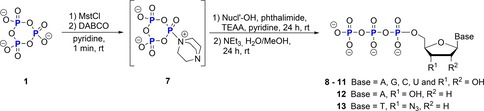
Synthesis of nucleoside triphosphates according to Mohamady and Taylor. MstCl=2‐mesitylensulfonyl chloride; DABCO=1,4‐diazabicyclo(2.2.2)octane; TEAA=triethylammonium acetate.

Mohamady and Taylor, who published a novel synthesis of nucleoside oligophosphates using cyclo‐TP **1** as triphosphorylation reagent, revived its application in nucleotide synthesis in the 2010s.^20^, ^33^ A first successful synthesis of protected nucleoside triphosphates by esterification was reported by the same group in 2016.20b The activation of **1** is split into two parts. First, it is sulfonylated with 2‐mesitylensulfonyl chloride (MstCl). Subsequently, they propose that the sulfonyl group is substituted in less than one minute by a nucleophilic attack of an amine base, such as 1,4‐diazabicyclo[2.2.2]octane (DABCO) or *N*‐methyl imidazole (NMI) to generate the reactive intermediate **7** that readily reacts with 2′,3′‐protected nucleosides (Scheme 4). Linearization with hydroxide is achieved in 100 mm triethylammonium acetate (TEAA) buffer (pH 7). After removal of protecting groups, followed by reverse phase HPLC (RP‐HPLC) purification, the target nucleoside triphosphates **8**–**13** were isolated in yields between 70 and 79 %.

**Scheme 5:**
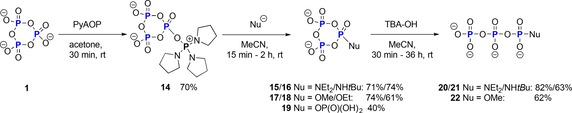
Synthesis of modified triphosphates according to Cummins. PyAOP=(7‐azabenzotriazol‐1‐yloxy)tripyrrolidinophosphonium hexafluorophosphate; TBA‐OH=tetrabutylammonium hydroxide; MeCN: acetonitrile.

In 2019, Cummins published a groundbreaking approach utilizing cyclo‐TP activation that enables reactions with C, N, and O nucleophiles.^21^ The bis(triphenylphosphine)iminium (PPN) salt of cyclo‐TP **1** reacts on a gram scale with the peptide coupling reagent PyAOP (Scheme 5) under ambient conditions to intermediate **14** in 70 % yield. In a second step, a nucleophile substitutes the tripyrrolidinophosphine oxide leaving group to give **15**–**19** in yields of 40 to 74 %. The modified cyclo‐TP is then hydrolyzed with tetrabutylammonium hydroxide (TBA‐OH), giving modified linear triphosphates **20**–**22** in yields between 54 and 82 %. Although this reagent has not yet been used with nucleosides, its reactivity with MeOH and EtOH (**17**, **18**) has been studied.

**Figure 3:**
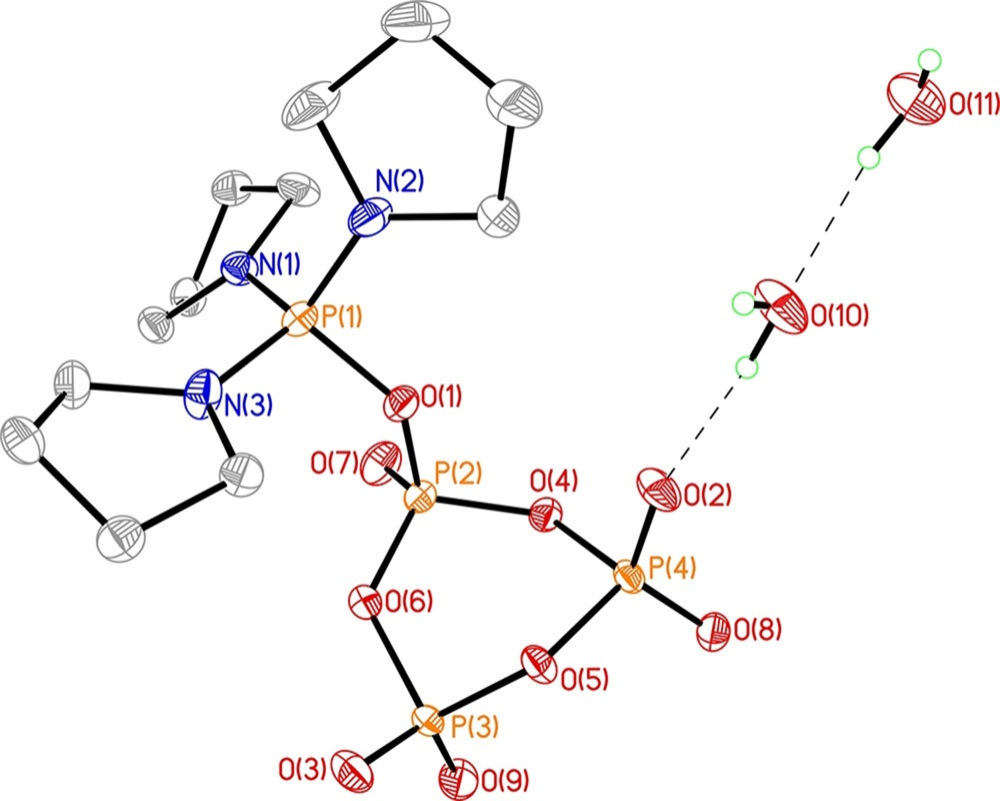
X‐ray structure of intermediate **14**. Reprinted with permission from S. M. Shepard, C. C. Cummins, *J. Am. Chem. Soc*. **2019**, *141*, 1852–1856. Copyright 2019 American Chemical Society.

Intriguingly, **14** is obtained as a stable solid that was studied by X‐ray crystal analysis (Figure 3) providing insight into its molecular structure. In the ^31^P NMR spectra, a peak at around −30 ppm is observed that corresponds to the central “ultraphosphate”‐like phosphorous atom of the reagent. The thorough characterization of the activated species will greatly facilitate the design of other reagents in the future and holds promise for further exciting discoveries.

**Scheme 6:**
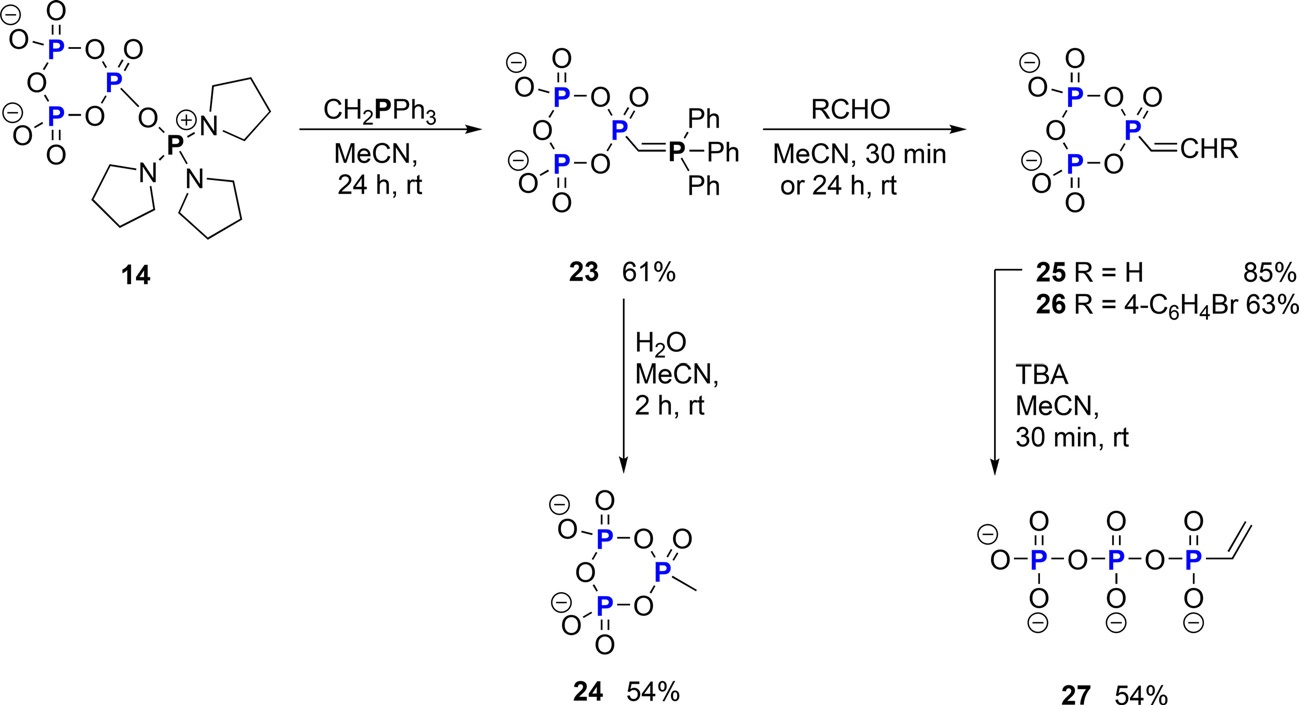
Reagent **14** is a versatile precursor to obtain triphosphate analogues containing a P−C bond.

The Cummins group goes on to demonstrate an extension of the work by using the Wittig reagent CH_2_PPh_3_, thereby including C‐nucleophiles in the reaction scope (Scheme 6). The resulting analogue **23** of cyclo‐TP was obtained in 61 % yield after 24 hours. With this product in hand, many different new manipulations can be envisioned. Simple hydrolysis yields a methyl phosphonate analogue **24** of cyclo‐TP. Reactions with aldehydes, however, enable the synthesis of alkenyl phosphonates **25** and **26** in yields between 63 and 85 %. Furthermore, the cyclic alkenyl phosphonate **25**, generated with formaldehyde, was linearized as described above to yield the first linear triphosphate analogue **27** synthesized from cyclo‐TP **1** with a P−C bond in 54 % yield.

**Scheme 7:**
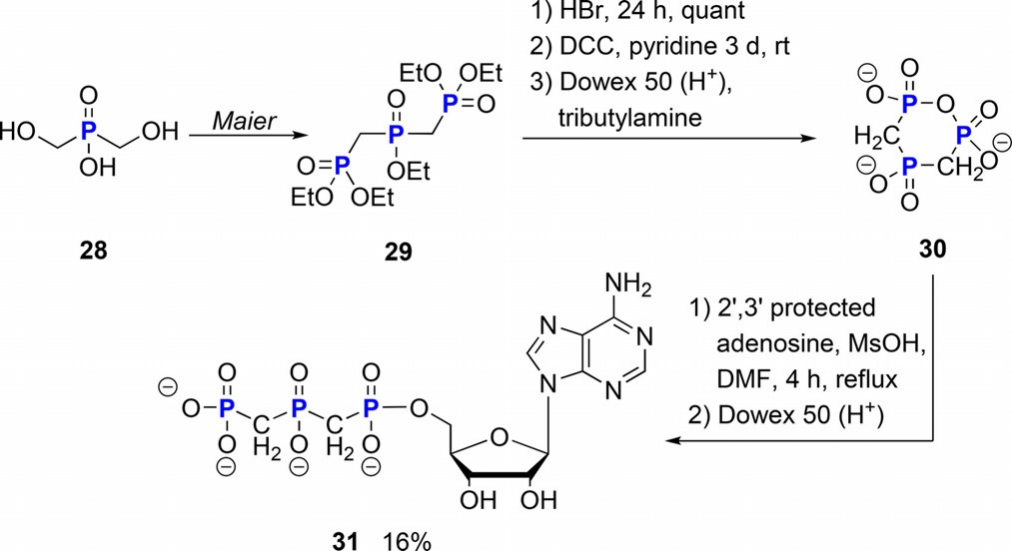
Synthesis of non‐hydrolyzable ATP **31** using the bismethylene analogue **30** of cyclo‐TP. MsOH = methanesulfonic acid; DMF = dimethylformamide.

In this context, the bismethylene analogue of cyclo‐TP **30** has been studied previously regarding its reactions with nucleophiles (Scheme 7). In 1969, Trowbridge reported the synthesis of a non‐hydrolyzable analogue **31** of ATP using the modified bismethylene cyclo‐TP **30** in 16 % yield.^12^ **29** was synthesized according to a procedure of Maier.^34^ These bismethylene analogues of cyclo‐TP have also been studied by Overhand^35^ as potential squalene synthase inhibitors. In these cases, however, the PCP bond was part of the ring structure, different to the compounds **23**–**27** reported by Cummins.

## 3 Synthesis of Cyclo‐TP Esters Using Phosphate–Ester Forming Reactions

**Scheme 8:**
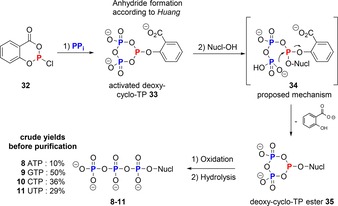
Synthesis of deoxy‐cyclo‐TP by P‐ester synthesis according to Huang.

There have been two reports on the formation of cyclo‐TP **1** from fragments of it that result in the generation of an activated electrophilic deoxy‐cyclo‐TP. The first approach by Huang is related to the Ludwig–Eckstein procedure, but reverses the order of addition of alcohol and pyrophosphate to reagent **32**. As a result, an activated deoxy‐cyclo‐TP **33** is formed (Scheme 8) that bears a phenolic leaving group on the P^III^ atom. This can then be replaced with nucleophiles in a two‐step mechanism proposed by Huang: Initially by attack on the P^III^ atom and cleavage of the anhydride, to give **34**, followed by regeneration of the deoxy‐cyclo‐TP ester **35** by expulsion of salicylate. This approach is applicable to unprotected nucleosides as demonstrated in their seminal study, resulting preferentially in triphosphorylation on the 5′‐OH (5′:3′ ratios ca. 85:15) giving products **8**–**11**.^36^

**Figure 4:**
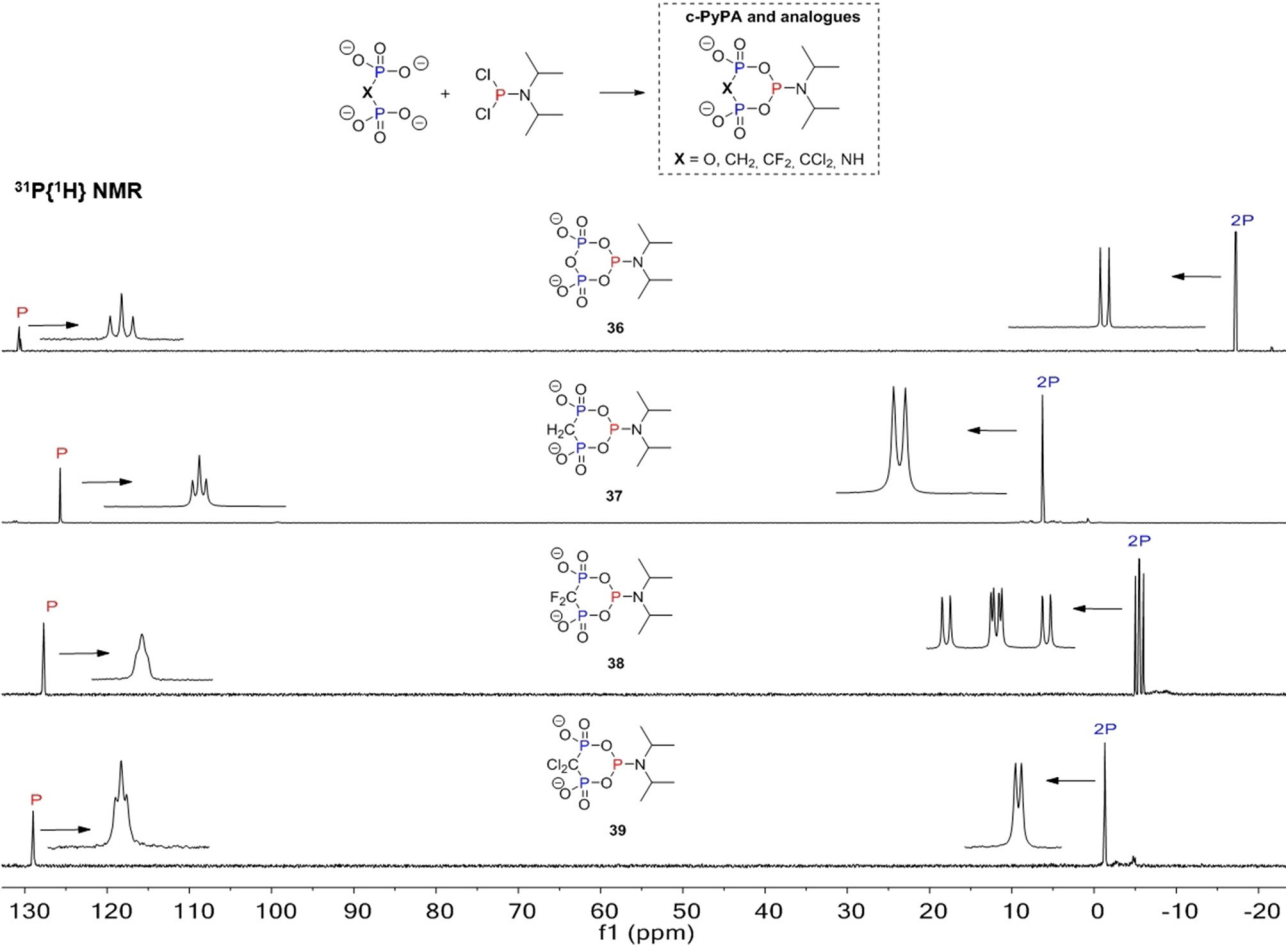
^31^P NMR (proton decoupled) analyses of cyclic pyrophosphoryl phosphoramidite **36** (c‐PyPA) and non‐hydrolysable analogues **37**–**39** in acetonitrile.

The second approach was published recently by our group in an effort to generate phosphoramidite (P‐amidite) analogues of **1**. The rationale behind this project was that phosphoramidites^37^ provide several benefits in terms of reactivity and selectivity, which we have exploited over the years in the selective construction of modified phosphoric anhydrides.^38^ Thus, it seemed promising to develop and explore such reagents in the context of tri‐ and polyphosphorylations. These reagents look similar to the activated deoxy‐cyclo‐TP **33** proposed by Huang (the P−O leaving group is exchanged for a P−N leaving group), but may have advantages regarding long‐term storability, high and tunable reactivity after activation (depending on the activator), and ease of handling. Intriguingly, also pyrophosphate analogues, such as several phosphonates can be used to generate a family of reagents, which are summarized in the dotted box in Figure 4 (cyclic pyrophosphoryl phosphoramidites, c‐PyPA **36** and analogues **37**–**39** with replacements of one oxygen atom).^39^ Figure 4 also shows the ^31^P NMR data of the reagents **36**–**39** generated in acetonitrile under dry conditions, underlining their stability, purity, and ease of preparation.

**Scheme 9:**
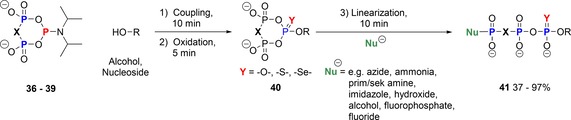
Synthesis of modified nucleoside triphosphate analogues with c‐PyPA reagents.

The reagents **36**–**39** were then applied to the synthesis of modified nucleoside triphosphates and non‐hydrolyzable analogues.39b After phosphitylation of alcohols with c‐PyPA reagents, the oxidation step enables the introduction of O, S, and Se in the α‐position. Detailed ^31^P NMR studies indicated very clean conversions, so that the linearization step could be conducted from almost pure cyclo‐TP esters **40**. Nucleophiles used in the linearization step included *N*‐nucleophiles (ammonia, azide, primary and secondary amines, imidazole), O‐nucleophiles (hydroxide, alcohols, fluorophosphate), and fluoride. In all cases, a clean linearization was observed, providing the products **41** in high yields, even in the case of unprotected nucleosides, such as T and A. In these cases partial phosphorylation also on the 3′‐OH was observed (ca. 10–15 %). Scheme 9 gives a general overview on the versatility of the approach.

DFT calculations provided insight into the mechanism of the ring‐opening reaction suggesting an in‐line attack of the nucleophile on the sterically least hindered phosphate followed by expulsion of the least charged leaving group. The proposed transition state is in line with the empirical reaction energetics leading to linearization and placement of the nucleophile in the terminal position.

## 4 Extension of the Concept: Synthesis of Phosphoric Anhydrides with Cyclo‐TP Analogues

**Scheme 10:**
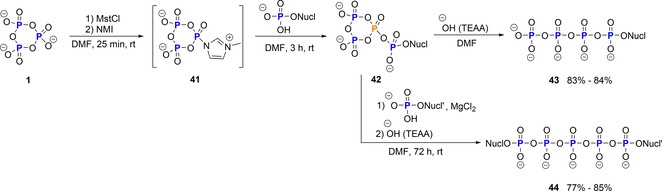
Synthesis of nucleoside tetraphosphates and dinucleoside pentaphosphates according to Mohamady and Taylor. NMI=*N*‐methyl imidazole.

Activated cyclo‐TP analogues can also be subjected to reactions with phosphate nucleophiles, thereby generating phosphorylated cyclo‐TPs like intermediate **42** that can be characterized as cyclic ultraphosphates, as they contain a trifurcation on at least one central phosphate subunit (Scheme 10, P at trifurcation highlighted in orange). In analogy to the previous chapters, such structures can also be linearized to give, for example, nucleoside tetraphosphates or linear polyphosphates.

Taylor has reported on the reaction of nucleoside monophosphates with their activated cyclo‐TP **41** (MstCl, followed by NMI), with subsequent linearization with hydroxide or other nucleotides to give nucleoside tetraphosphates **43** and dinucleoside pentaphosphates **44**, respectively (Scheme 10).20a Additionally, fluorescent labels were attached to nucleoside tetraphosphates using this strategy.^33^ Recently, Kool has made use of Taylor's approach to synthesize dicaptides, which are mixed dinucleoside pentaphosphates. These are substrates of the Klenow fragment of DNA polymerase I and other polymerases. Interestingly, two of these heterodimeric nucleotides suffice for full four‐base primer extension on DNA template strands.^40^

**Scheme 11:**
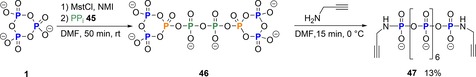
Synthesis of linear octaphosphate with terminal propargyl phosphoramidates P‐amidates.

This approach by Taylor was also used in our laboratory to construct the first monodisperse linear octaphosphate analogue with terminal phosphoramidate labels for polyphosphate transfer studies to proteins. The study demonstrated that cyclo‐TP **1** is an excellent precursor to build up longer polyphosphate chains by bidirectional synthesis.^41^ It proceeds by reacting two equivalents of the activated cyclo‐TP with pyrophosphate **45** giving the pyrophosphate bridged cyclo‐TP **46** as intermediate (Scheme 11) that is then linearized with amine nucleophiles. The symmetric intermediate was identified by ^31^P NMR in solution and the product **47** with terminal propargyl phosphoramidates was obtained in 13 % isolated yield after ion exchange chromatography.

**Scheme 12:**
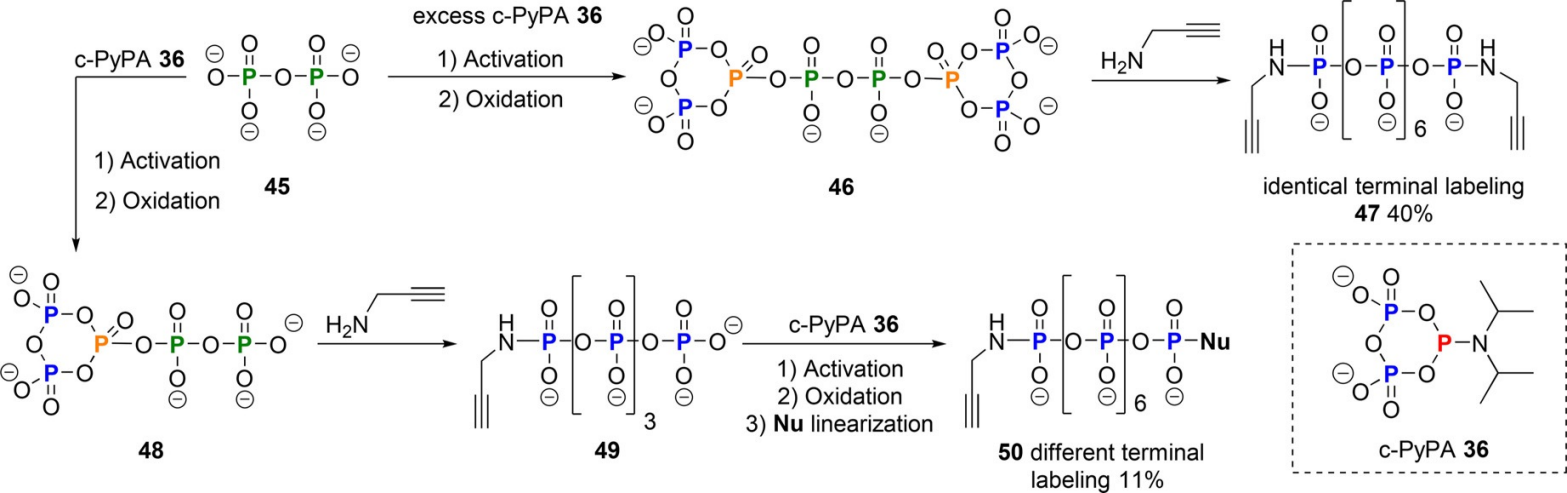
Synthesis of linear octaphosphate with identical or different terminal labeling using c‐PyPA **36**.

The need to access polyphosphate probes with defined chain length and modifications for studies into its biological functions has also motivated a follow‐up study using c‐PyPA **36** as triphosphorylating reagent.39a This application has led to significantly improved yields (40 % of **47**) and also enabled the generation of polyphosphates with different labels at the chain termini as shown in Scheme 12. In brief, c‐PyPA **36** was reacted in excess with pyrophosphate **45** to achieve reaction on both terminal phosphates. The P^III^ intermediate was oxidized with *m*CPBA to give the same intermediate **46** as previously achieved with Taylor's reagent. Linearization with propargylamine gave an eight‐units polyphosphate **47** with identical phosphoramidates on the termini. On the other hand, the use of c‐PyPA **36** in limiting amounts enabled the selective reaction with pyrophosphate **45** on one terminus only, giving access to intermediate **48**, as demonstrated by ^31^P NMR spectroscopy. After linearization with propargylamine, the five‐units polyphosphate **49** with one terminal phosphoramidate was obtained. The unreacted terminus of that compound easily engaged in a second round of coupling with c‐PyPA **36**, followed by oxidation and ring‐opening with a second nucleophile. This iterative triphosphorylation strategy using **36** provides access to polyphosphates with different labels at the termini (**50**, Scheme 12). Such compounds will be useful in studying polyP turnover in cells^42^ and could potentially help in identifying the elusive mammalian polyP kinase.

Cummins has reported on the application of their PyAOP‐activated cyclo‐TP reagent **14** in reactions with phosphate as a nucleophile to generate monophosphorylated cyclo‐TP **19** (see Scheme 5). The identity of this intriguing structure was confirmed by X‐ray crystallography.^21^ Thus, **14** can also be used to construct efficiently phosphoric anhydrides.

**Scheme 13:**
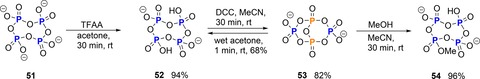
Condensation chemistry of cyclotetraphosphate and synthesis of its monoesters. TFAA=trifluoroacetic anhydride.

To extend the applications of cyclo‐TP **1** and improve our general understanding of cyclophosphate chemistry, larger cyclic phosphates and their reactions are under investigation. Cummins, for example, reported a modification of cyclotetraphosphate **51** (tetrametaphosphate) to synthesize the first fully characterized esters of this family as shown in Scheme 13.^43^ Tetrametaphosphate gets protonated in presence of a strong acid like trifluoroacetic acid (TFA) to generate dihydrogen tetrametaphosphate **52** in 94 % yield on a gram scale. Further treatment with *N*,*N*′‐dicyclohexylcarbodiimide (DCC) resulted in a condensed intermediate **53** that is in equilibrium with dihydrogen tetrametaphosphate **52**. This intermediate contains an “ultraphosphate‐type” structure with characteristic shifts in the ^31^P NMR at around −35 ppm, featuring a bicyclic trimetaphosphate with a phosphoric anhydride bridge.^44^ Its structure was established by X‐ray diffraction analysis. The forward reaction was carried out in acetonitrile with a yield of 82 %. The backward reaction occurred in wet acetone within one minute to regenerate the dihydrogen form in 68 % isolated yield. Methanol was used as nucleophile to cleave the anhydride and form a novel methyl cyclotetraphosphate ester **54** in 96 % yield.

## Outlook

The generation of “ultraphosphate‐type” intermediates shown in Scheme 5, Scheme 10, Scheme 11 and Scheme 12 by a nucleophilic attack on activated cyclo‐TP promises efficient entry to the underexplored field of branched condensed phosphates devoid of cyclic substructures. Currently, ultraphosphates are generally known as polydisperse glass‐forming and crystalline substances with a variety of substructures.^45^ Already in the 1950s, cross‐links within phosphate chains were detected after heating NaHPO_4_⋅H_2_O to 970 °C for 14 h^46^ and initial data on the hydrolysis of the branching points were collected by viscosity and pH measurements.^47^ Although crystalline ultraphosphates have been used in a range of different applications by high‐temperature processes,^48^ no rational synthesis of defined molecular non‐cyclic ultraphosphates has been reported so far. Moreover, considering that all possible classes of condensed phosphates have been identified in biology, the absence of ultraphosphates in this list is surprising. Yet, the idea of their potential involvement in biological processes has been discarded and will be experimentally demanding to study “because they are unusually rapidly hydrolyzed in aqueous solution”.14c Particularly in light of the lack of data on monodisperse ultraphosphates regarding their hydrolysis half‐life and the fact that unstable structures occur as intermediates in biology, a synthetic access to well‐defined ultraphosphates to better understand their properties is desirable and may lead to interesting new findings.

**Scheme 14:**
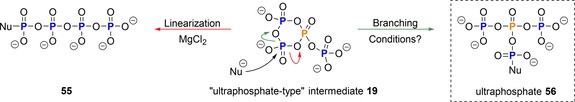
Possible formation of ultraphosphates by directed ring‐opening using the “ultraphosphate‐type” intermediate derived from cyclo‐TP **1**.

The synthesis of defined structures could, for example, commence from cyclo‐TP analogues as described above (Scheme 14),^21^, ^41^ if the regioselectivity of the ring‐opening on the ultraphosphate‐like intermediate **19** could be controlled. Up to now, only linearized products **55** were isolated after the attack of a nucleophile on cyclo‐TP anhydrides39a and DFT calculations suggest a difference of about 8 kcal mol^−1^ in the transition states preferring linearization over branching.39b Nevertheless, various conditions and metal coordination may be exploited to direct the ring‐opening towards branching. In doing so, several modifications may be introduced by using different nucleophiles to give **56** facilitating in vitro or in vivo studies with novel ultraphosphate tools to study the occurrence of ultraphosphates in biology. Moreover, ultraphosphates could—as high‐energy entities—represent an underappreciated phosphorylating agent in prebiotic chemistry—much more reactive than for example, cyclo‐TP **1** itself.

The synthesis of larger cyclophosphate rings and their transformation into novel reagents is also a highly promising area of research. One recent example is the cyclic ultraphosphate disclosed by Cummins (see Scheme 13). Many more reagents of this type can be envisioned—as structurally beautiful and at the same time highly useful compounds for manifold applications.

## Conclusions

The direct customization of cyclo‐TP **1** or its assembly from precursors as reagents in the synthesis of diverse condensed phosphates is setting up significant possibilities in the field of nucleotide synthesis, but also beyond. These compounds have interesting implications and applications in the fields of prebiotic chemistry, polyelectrolyte research, and as reagents in organic and inorganic synthesis. Modified (cyclic)polyphosphates are now becoming accessible, and the potential generation and study of defined ultraphosphates and their interactions with metals holds promise to open up a whole new area of research that has only received very limited attention in recent years. Such studies could further lead to new concepts in prebiotic phosphorylation chemistry and will in general provide new insights into the field of the condensed phosphates.

2019 is the year of the periodic table and also the 350th anniversary of the discovery of elemental phosphorous by Hennig Brand in Hamburg. We hope that with this perspective, we have not only informed about recent developments in cyclo‐TP chemistry, but also conferred to the reader—at least in part—our fascination of the condensed phosphates—a substance class that provides so many challenges and opportunities still waiting to be explored.

## Conflict of interest

The authors declare no conflict of interest.
